# Strict maternal inheritance of chloroplast and mitochondrial genomes in *Bryum argenteum*

**DOI:** 10.1186/s12870-026-09627-y

**Published:** 2026-07-31

**Authors:** Xiangxi He, Fengjun Leng, Yong Hu, Yikun He

**Affiliations:** Key Laboratory of Plant Gene Resources and Biotechnology for Carbon Reduction and Environmental Improvement, Beijing Municipal Government, and College of Life Sciences, Capital Normal University, Beijing, 100048 China

**Keywords:** Moss, *Bryum argenteum*, Chloroplast genome, Mitochondrial genome, Maternal inheritance, Paternal leakage

## Abstract

Organelle inheritance is predominantly maternal in most eukaryotes, yet quantitative estimates of paternal leakage—the low-frequency transmission of paternal cytoplasmic genomes—remain scarce outside seed plants. In bryophytes, the sister group to tracheophytes, maternal inheritance of chloroplasts and mitochondria has been confirmed qualitatively in a small number of species, but no study has quantified paternal leakage rates. We assembled the complete chloroplast (122,995 bp) and mitochondrial (101,373 bp) genomes of *Bryum argenteum* from a Beijing population. An intraspecific cross was performed between an Antarctic maternal genotype(ma1) and a Beijing paternal genotype(XA), generating seven independent capsules from which 21 F₁ gametophytes were deep-sequenced. De novo assembly of all 21 F₁ individuals recovered exclusively the maternal haplotype at all 464 chloroplast and 96 mitochondrial diagnostic sites. Quantitative allele frequency analysis of 363 chloroplast and 58 mitochondrial diagnostic SNPs across 8,841 independent tests detected no paternal-specific allele exceeding empirically defined noise thresholds(99th percentiles of maternal background: 2.50% for chloroplasts, 2.25% for mitochondria). Principal component analysis of 421 diagnostic SNPs clustered all F₁ individuals with the maternal parent (PC1 = 98.1% of variance). The paternal leakage frequency was conservatively estimated at < 0.1% (upper 95% confidence limit 0.034%). Slightly elevated paternal-type mitochondrial signals in F₁ progeny (median 0.18% vs. maternal background 0.04%; *p* = 0.042) were attributable to the faithful transmission of pre-existing maternal heteroplasmy, demonstrated by a strong maternal-F₁ correlation (Pearson's *r* = 0.643, *p* = 7.31 × 10⁻5, R2 = 0.413). These results deliver quantitative evidence for conserved uniparental organelle transmission in a non-seed plant, underscoring the ancestral nature of maternal inheritance during land plant evolution.

**Supplementary Information:**

The online version contains supplementary material available at https://doi.org/10.1186/s12870-026-09627-y.

## Background

Organelle inheritance is predominantly uniparental and follows the maternal line in most animals and angiosperms, whereas in gymnosperms paternal inheritance is the rule [1]. An increasing number of studies reveal that even in species with strict maternal inheritance, paternal leakage—the low-frequency transmission of paternal organelles to progeny—occurs and is not a rare phenomenon [2, 3].

Quantitative estimates of paternal leakage rates have been obtained in several plant systems. In tobacco (*Nicotiana tabacum*), the frequency of paternal mitochondrial transmission under normal conditions is approximately 0.18%; however, knockout of the exonuclease *DPD1* or chilling stress during pollen development can increase this frequency dramatically, to 7.34% [4]. This directly demonstrates that specific genetic defects and environmental stress can destabilize the maternal inheritance barrier. In natural populations of *Silene vulgaris* and *Plantago lanceolata*, mitochondrial heteroplasmy caused by paternal leakage has been detected, indicating that the phenomenon is geographically widespread [5, 6]. In inter-lineage hybrids of *Silene nutans*, up to 98% of surviving progeny inherited the paternal plastid genome—interpreted as a rescue mechanism when the maternal plastid is incompatible with the hybrid nuclear genome [2]. In *Passiflora*, 45 interspecific crosses revealed clade-specific plastid inheritance patterns, with biparental inheritance restricted to early developmental stages [7].

In animals, paternal leakage has been documented across diverse taxa: a single event among 385 transmissions in a 50-generation chicken pedigree [8]; paternal mtDNA in natural loach populations via high-throughput sequencing [9]; varying proportions of maternal and paternal mtDNA in sea turtle hybrids [10]; up to 59% of copepod F₁ hybrids carrying both maternal and paternal mtDNA [11]; and mtDNA heteroplasmy detected in tick experimental crosses [12]. Together, these findings establish paternal leakage as a widespread phenomenon across plants and animals.

Current detection methods—parent-specific SNP markers, deep sequencing, allele-specific qPCR, and digital droplet PCR (ddPCR)—achieve sensitivities approaching 0.01% for low-frequency heteroplasmy [13]. However, reliable estimation requires correction for several confounding factors: nuclear mitochondrial pseudogenes (NUMTs) can masquerade as paternal leakage signals [14, 15]; intrinsic low-level maternal heteroplasmy can be transmitted to offspring and mistaken for paternal leakage [16]; and inadequate sequencing depth, tissue-specific variation, and PCR bias affect reliability [17, 18]. A rigorous framework therefore requires background noise characterization, parent-specific markers, large-sample replication, and statistical upper-bound calculation [19].

Research on organelle inheritance has focused predominantly on seed plants [1]. In bryophytes—the sister group to tracheophytes and thus occupying a key phylogenetic position for understanding organelle inheritance across land plants [20]—only four studies have examined organelle transmission. Pacak & Szweykowska-Kulińska (2003) demonstrated uniparental organellar inheritance in the allopolyploid liverwort *Pellia borealis* using organellar intron markers across 14 populations [21]. Jankowiak et al. (2005) showed maternal transmission of both chloroplasts and mitochondria in the allopolyploid moss *Rhizomnium pseudopunctatum* [22]. McDaniel et al. (2007) provided the first experimental confirmation via controlled crosses, demonstrating that all ten recombinant haploid progeny from a *Ceratodon purpureus* interpopulation cross exclusively carried the maternal chloroplast haplotype [23]. Natcheva & Cronberg (2007) confirmed strict maternal inheritance of both organelles in interspecific *Sphagnum* hybrids [24]. Critically, none of these studies attempted to quantify paternal leakage rates. Among an estimated 18,000–25,000 bryophyte species worldwide, the quantitative dimension of organelle inheritance remains entirely unexplored.

Quantifying paternal leakage in bryophytes is important for several reasons. First, genetic elements with uniparental inheritance are disproportionately involved in reproductive isolation, as cytonuclear incompatibilities can arise rapidly and generate asymmetric postmating barriers [25]. Second, the molecular mechanisms enforcing uniparental transmission—including degradation of paternal organelles during fertilization—remain poorly understood outside a few angiosperm models. Bryophytes, with their dominant haploid gametophyte phase and external fertilization, offer a distinct developmental context for dissecting these mechanisms. Third, establishing a baseline paternal leakage rate in a moss provides a reference point for testing whether environmental or genetic perturbations can destabilize maternal inheritance, as recently demonstrated in tobacco [4].

*Bryum argenteum* is a cosmopolitan moss species occurring from Antarctica to urban habitats [26, 27], tolerant of desiccation, extreme temperatures, and heavy metal pollution [26, 28]. Its relatively small genome (~ 313 Mb), amenability to laboratory cultivation, and the recent development of PEG-mediated genetic transformation [29] make it an emerging model for functional studies in bryophytes. Here, we assembled the complete organelle genomes of *B. argenteum*, performed controlled intraspecific crosses between geographically distant parental genotypes, and conducted the first quantitative assessment of paternal leakage frequency in a bryophyte. We also present an analytical framework—incorporating empirical noise thresholding, maternal heteroplasmy correction, and binomial confidence bounding—that is broadly applicable to organelle inheritance studies in non-seed plants.

## Materials and methods

### Sample collection, culture, and DNA extraction

The maternal parent (ma1) was collected from Marble Point, Ross Sea, Antarctica. The paternal parent (XA) was collected from the Capital Normal University campus, Beijing, China. Both were morphologically identified by Prof. Yikun He. Axenic cultures were established following the sterilization protocol of Leng et al. [29] and maintained on half-strength Murashige and Skoog (MS) solid medium at 20 ± 1 °C under a 16:8 h light:dark photoperiod (40 μmol·m⁻2·s⁻1). Voucher specimens were deposited in the Beijing Capital Normal University Herbarium (BJTC; ma1: BJTC014089; XA: BJTC014074).

Total genomic DNA was extracted from approximately 50 mg of fresh cultured tissue using a modified CTAB method. Library construction (350 bp insert) and paired-end 150 bp sequencing were performed on the Illumina HiSeq 2500 platform. Raw reads were quality-filtered using fastp (v0.23.2) [30].

### Organelle genome assembly and annotation

Chloroplast and mitochondrial genomes were assembled using GetOrganelle (v1.7.7.1) [31]. Assembly graphs were inspected with Bandage [32]. Genome maps were drawn using OGDRAW v1.3.1 [33]. Annotations were performed using GeSeq [34] and MITOS2 [35, 36] with manual curation against published bryophyte organelle genomes.

### Identification of diagnostic markers between parents

The maternal (ma1) and paternal (XA) organelle genomes were aligned using MAFFT (v7.505) [37] to identify diagnostic sites. SNPs and indels were detected by mapping reads to the XA reference using BWA-MEM (v2.2.1) [38, 39] followed by variant calling with GATK Mutect2 (v4.4.0.0) [40, 41] and filtering (Q ≥ 20) using Bcftools (v1.10.2) and Samtools (v1.17) [42, 43].

### Hybridization and progeny sequencing

Stem tips of female (ma1) and male (XA) gametophytes were co-cultivated in autoclaved glass bottles containing sterilized soil-vermiculite mixture (1:1, v/v) under 16:8 h light:dark at 22 °C. Seven independent sporophytes matured; spores from each capsule were collected and germinated on half-strength MS medium. Three F₁ gametophytes with consistent growth from each capsule (21 individuals total) were selected for genomic DNA extraction and deep sequencing.

### Analysis of paternal leakage

F₁ sequencing reads were mapped to both maternal (ma1) and paternal (XA) organelle reference sequences using BWA-MEM. After sorting and deduplication (Samtools), variant calling was performed with GATK Mutect2. Only variants with ≥ 200 × depth and present in ≥ 2 F₁ individuals were retained.

Quantitative allele frequency analysis targeted 361 chloroplast and 60 mitochondrial diagnostic SNPs (indels were excluded as unsuitable for low-frequency allele quantification). Allele frequencies were calculated from the Mutect2 output.

### Empirical noise threshold determination

To establish rigorous detection thresholds, we first characterized the technical noise distribution by mapping paternal XA reads against the XA reference (self-mapping). The 99th percentile of non-reference allele frequencies in self-mapping was 1.5%, defined as the baseline technical noise ceiling attributable to sequencing error and alignment artifacts. Cross-mapping of maternal ma1 reads against the XA reference resolved three frequency classes: (i) AF < 1.5%, consistent with sequencing noise; (ii) AF 1.5–5%, representing systematic mapping bias (cross-genotype reference bias) mixed with very low-level biological variation; and (iii) AF > 5%, representing true maternal heteroplasmy (Fig. 2a, b). The 5% boundary was chosen because self-mapping signals never exceeded ~ 3%, and cross-mapping AF > 5% were significantly above this maximum (binomial test, p < 0.001, corrected for sequencing depth).

We then measured the natural occurrence of paternal diagnostic alleles in the maternal background specifically at the 361 chloroplast and 60 mitochondrial diagnostic SNPs. Paternal-type allele frequencies at these sites ranged from 0–2.50% (chloroplast) and 0–2.25% (mitochondria). The 99th percentiles of these distributions (2.50% for chloroplasts, 2.25% for mitochondria) were adopted as empirical detection thresholds (Fig. 2c). These are far more conservative than the 1.5% baseline noise level, ensuring that conclusions are robust to the exact placement of intermediate classification cutoffs. We note that these thresholds are calibrated to our ~ 1000 × sequencing depth and may require re-calibration for different coverage levels.

### Statistical framework

Following the binomial principle that when zero events are observed in N independent tests, the upper 95% confidence limit for the true event frequency is approximately 3/N [19], we calculated an upper bound of 0.034% (*N* = 8,841 = 361 × 21 + 60 × 21). To provide a conservative estimate that accommodates potential biases from variable sequencing depth across loci, uncorrected technical artifacts, and facilitates cross-study comparison with animal mitochondrial paternal leakage estimates (commonly reported at the 0.1% level [3, 15]), we report the paternal leakage frequency as ≤ 0.1%.

### Principal component analysis

PCA was performed on the paternal allele frequency matrix of 421 diagnostic SNPs (361 chloroplast + 60 mitochondrial) across 23 samples (ma1, XA, and 21 F₁ progeny) using R. The PCA provides a multivariate summary of organelle SNP profile similarity.

## Results

### Complete organelle genomes of *B. argenteum*

**Fig. 1:**
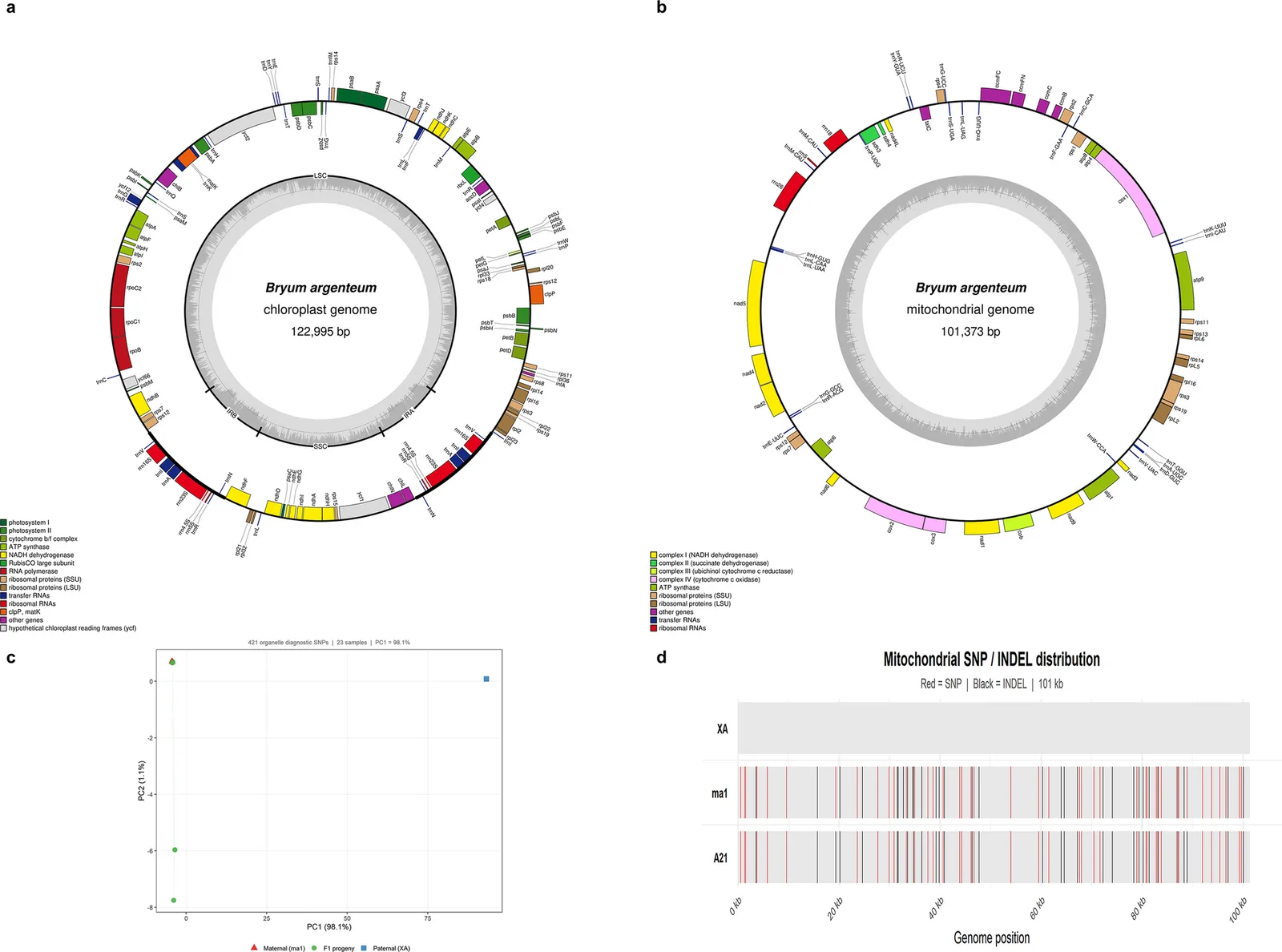
Genomic resources and diagnostic markers. **a** Chloroplast genome of *B. argenteum* (XA reference). Outer ring: forward-strand genes (color-coded by function); inner ring: GC content (500-bp window). IR, inverted repeat; LSC, large single copy; SSC, small single copy. **b** Mitochondrial genome of XA. Genes are color-coded according to respiratory complexes (I–V), ribosomal proteins, rRNAs, and tRNAs. Inner circle: GC content. **c** Principal component analysis of organelle diagnostic SNP profiles. PCA of paternal allele frequencies across 421 diagnostic SNPs (361 chloroplast + 60 mitochondrial) for 23 samples. All 21 F₁ individuals (green) cluster tightly with the maternal parent ma1 (red) and are clearly separated from the paternal parent XA (blue). PC1 explains 98.1% of total variance. **d** Distribution of mitochondrial SNPs and indels along the genome. Three tracks (top to bottom): paternal XA reference, maternal ma1, and a representative F₁ individual (A21). Grey bars: reference background; red vertical lines: SNPs; black vertical lines: indels. The F₁ pattern is identical to the maternal ma1 pattern, confirming strict maternal inheritance at single-nucleotide resolution

We assembled the complete organelle genomes of the Beijing reference population (XA): a 122,995 bp chloroplast genome (GenBank PZ276161) and a 101,373 bp mitochondrial genome (GenBank PZ276158). The chloroplast genome exhibits the typical quadripartite architecture: large single-copy region (LSC, 85,591 bp), small single-copy region (SSC, 18,422 bp), and two inverted repeats (IR, 9,491 bp each). Overall GC content is 28.07%, with markedly elevated GC content in the IR regions (43.76%) relative to single-copy regions (LSC 24.58%, SSC 25.35%). The genome encodes 123 genes: 81 protein-coding, 34 tRNAs, and 8 rRNAs, including complete suites of photosynthesis-related genes (*psa/psb, atp, ndh, pet, rbcL*) and ribosomal proteins (12 *rps*, 10 *rpl*). Twenty-four genes contain introns: 11 protein-coding and 5 tRNA genes harbor a single intron, whereas *ycf3, rps12*, and *clpP* each contain two introns (Table 1, Fig. 1a).

**Table 1:** Gene composition of the *B. argenteum* chloroplast genome (XA reference)

Category of genes	Group of genes	Name of genes
Genes for photosynthesis	Subunits of ATP synthase	*atpB, atpE, atpA, atpF, atpH, atpI*
	Subunits of protochlorophyllide reductase	*chlB, chlN, chlL*
	Subunits of photosystemII	*psbH, psbN, psbT, psbB, psbE, psbF, psbL, psbJ, psbZ, psbC, psbD, psbA, psbK, psbI, psbM*
	Subunits of NADH-dehydrogenase	*ndhC, ndhK, ndhJ, ndhB, ndhF, ndhD, ndhE, ndhG, ndhI, ndhA, ndhH*
	Subunits of cytochromeb/f complex	*petD, petB, petG, petL, petA*
	Subunits of photosystemI	*psaJ, psaI, psaA, psaB, psaM, psaC*
	Subunit of rubisco	*rbcL*
Self replication	Large subunit of ribosome	*rpl23, rpl2, rpl22, rpl16, rpl14, rpl36, rpl20, rpl33, rpl21, rpl32*
	DNA dependent RNA polymerase	*rpoC2, rpoC1, rpoB*
	Small subunit of ribosome	*rps19, rps3, rps8, rps11, rps12, rps18, rps4, rps14, rps2, rps7, rps15*
rRNA	Ribosomal RNAs	*rrn16S,rrn23S,rrn4.5S,rrn5S*
tRNA	Transfer RNAs	*trnI(3), trnP, trnW, trnR(4), trnM, trnF, trnL(2), trnT(2), trnS(3), trnfM, trnG(2), trnE, trnY, trnD, trnH, trnK, trnQ, trnC, trnV(2), trnA(2), trnN(2)*
Other genes	Subunitof Acetyl-CoA-carboxylase	*accD*
	Maturase	*matK*
	Conserved open reading frames	*ycf4, ycf3##, ycf2#, ycf12, ycf66#, ycf1*
	Protease	*clpP*
	*Translational initiation factor*	*infA*

The mitochondrial genome is a single circular molecule with a GC content of 39.25%. It contains 65 functional genes: 38 protein-coding, 3 rRNAs (*rrn5, rrn18, rrn26*), and 24 tRNAs (Table 2). Protein-coding genes represent all five respiratory chain complexes, including 14 ribosomal proteins. Fourteen genes contain introns; *cox1* harbors four introns, *atp9, cox2*, and *nad5* each contain three (Fig. 1b). The organelle genome sizes and gene contents are consistent with those reported for other mosses, reflecting the well-documented structural conservation of bryophyte organelle genomes [44–50].

**Table 2:** Gene composition of the *B. argenteum* mitochondrial genome (XA reference)

Category	Gene Group	Gene Name
	Subunits of ATP synthase	*atp9###, atp4, atp8, atp6#, atp1#*
Self-replication	Proteins of large ribosomal subunit	*rpL2, rpl16, rpL5, rpL6*
	Proteins of small ribosomal subunit	*rps1, rps2, rps4, rps12, rps7, rps19, rps3, rps14, rps13, rps11*
rRNA	Ribosomal RNAs	*rrn18, rrn5, rrn26*
tRNA	Transfer RNAs	*trnI-CAU, trnK-UUU, trnC-GCA, trnF-GAA, trnQ-UUG, trnL-UAG, trnS-UGA, trnG-UCC, trnR-UCU, trnY-GUA, trnP-UGG, trnM-CAU(2), trnH-GUG, trnL-CAA, trnL-UAA, trnG-GCC, trnR-ACG, trnE-UUC, trnW-CCA, trnV-UAC, trnD-GUC, trnA-UGC, trnT-GGU*
	Other	*cox1####, ccmB, ccmC, ccmFN, ccmFC#, tatC, nad4L, sdh4, sdh3#, nad5###, nad4#, nad2#, nad6, cox2###, cox3#, nad1##, cob#, nad9#, nad3*

### Diagnostic markers between parental genotypes

Comparison of the maternal (ma1) and paternal (XA) organelle genomes identified 464 differential sites in the chloroplast (361 SNPs and 103 indels) and 96 in the mitochondria (60 SNPs and 36 indels). This substantial divergence—reflecting the intercontinental separation of the parental populations (Antarctica vs. Beijing)—provided abundant diagnostic markers for tracking organelle transmission in controlled crosses. The mitochondrial SNP and indel distribution along the genome, shown for the paternal reference (XA), maternal parent (ma1), and a representative F₁ individual (A21), confirms that F₁ patterns are identical to the maternal parent at single-nucleotide resolution (Fig. 1d).

### Hybridization confirms maternal inheritance at the assembly and multivariate levels

Seven independent capsules were generated using ma1 as the maternal and XA as the paternal parent. Twenty-one F₁ gametophytes (three per capsule) were deep-sequenced (> 1000 ×). De novo assembly of all 21 F₁ individuals recovered exclusively the maternal haplotype at both organelle genomes, with F₁ sequences matching ma1 at all 464 chloroplast and 96 mitochondrial diagnostic sites. No paternal XA haplotypes were detected in any F₁ individual at the assembly level.

To provide a multivariate summary of these results, we performed principal component analysis of paternal allele frequencies across all 421 diagnostic SNPs. All 21 F₁ individuals clustered tightly with the maternal parent ma1 (PC1 explaining 98.1% of total variance) and were clearly separated from the paternal parent XA (Fig. 1c). This provides a visually immediate confirmation of exclusive maternal inheritance, complementing the quantitative allele frequency analyses below.

### Quantitative noise modeling and detection thresholds

**Fig. 2:**
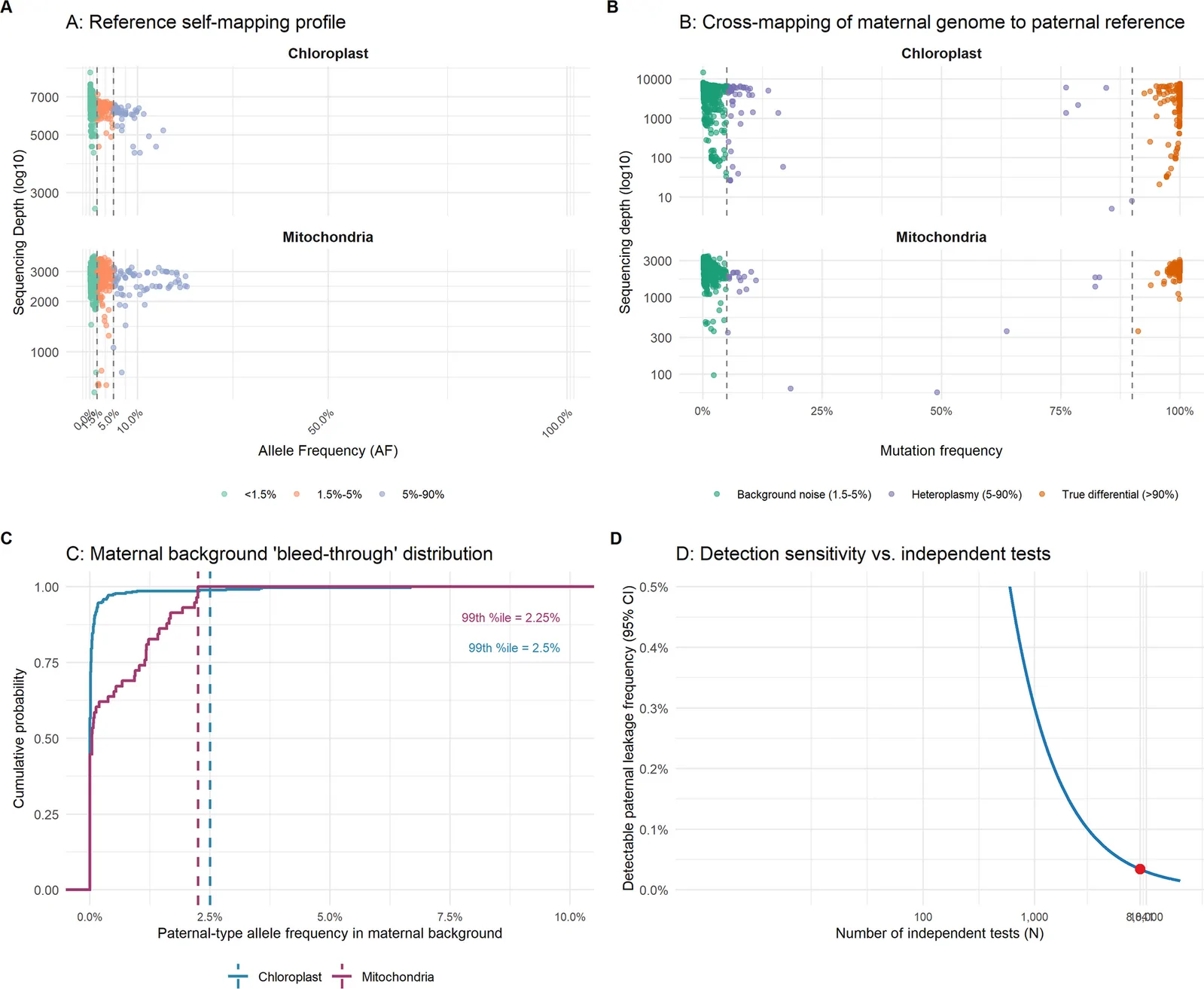
Experimental noise modeling. **a** XA self-mapping (paternal reads aligned to the paternal reference). Points are colored by allele frequency (AF) category: AF < 1.5% (green, baseline technical noise), 1.5–5% (orange, systematic mapping bias), and > 5% (purple/pink). **b** Cross-mapping of maternal ma1 reads onto the paternal XA reference. Three categories: AF > 90% (red, true differential sites), 5–90% (purple, candidate heteroplasmy), and 1.5–5% (green, biological background noise). **c** Cumulative distribution of paternal-type allele frequencies at diagnostic SNPs in the maternal background (ma1). Dashed lines: 99th percentile thresholds (2.50% for chloroplasts, 2.25% for mitochondria). **d** Detection sensitivity curve (upper 95% confidence limit = 3/N). Red point: this study (*N* = 8,841, upper bound = 0.034%)

To establish a rigorous baseline for detecting rare paternal transmissions, we first quantified technical noise by self-mapping paternal XA reads to the XA reference. This revealed low-frequency signals at 153 chloroplast and 718 mitochondrial sites. Allele frequencies below 1.5% were defined as baseline technical noise, whereas signals between 1.5% and 5% were attributed to systematic mapping bias (Fig. 2a).

Cross-mapping of maternal ma1 reads onto the paternal XA reference resolved three signal classes: (i) AF > 90%, representing true differential sites; (ii) AF 5–90%, representing candidate maternal heteroplasmy (43 chloroplast and 17 mitochondrial sites); and (iii) AF 1.5–5%, representing biological background noise from cross-genotype mapping (135 chloroplast and 282 mitochondrial sites) (Fig. 2b).

We further measured the natural occurrence of paternal diagnostic alleles in the maternal background. At the 361 chloroplast and 60 mitochondrial diagnostic SNPs, paternal-type allele frequencies in ma1 ranged from 0–2.50% and 0–2.25%, respectively. The 99th percentiles of these distributions (2.50% for chloroplasts, 2.25% for mitochondria) were adopted as empirical detection thresholds (Fig. 2c). Based on these thresholds and 8,841 independent tests, the 95% confidence upper limit for detectable paternal leakage was 0.034% (Fig. 2d).

### Quantitative analysis reveals no paternal leakage in F₁ progeny

**Fig. 3:**
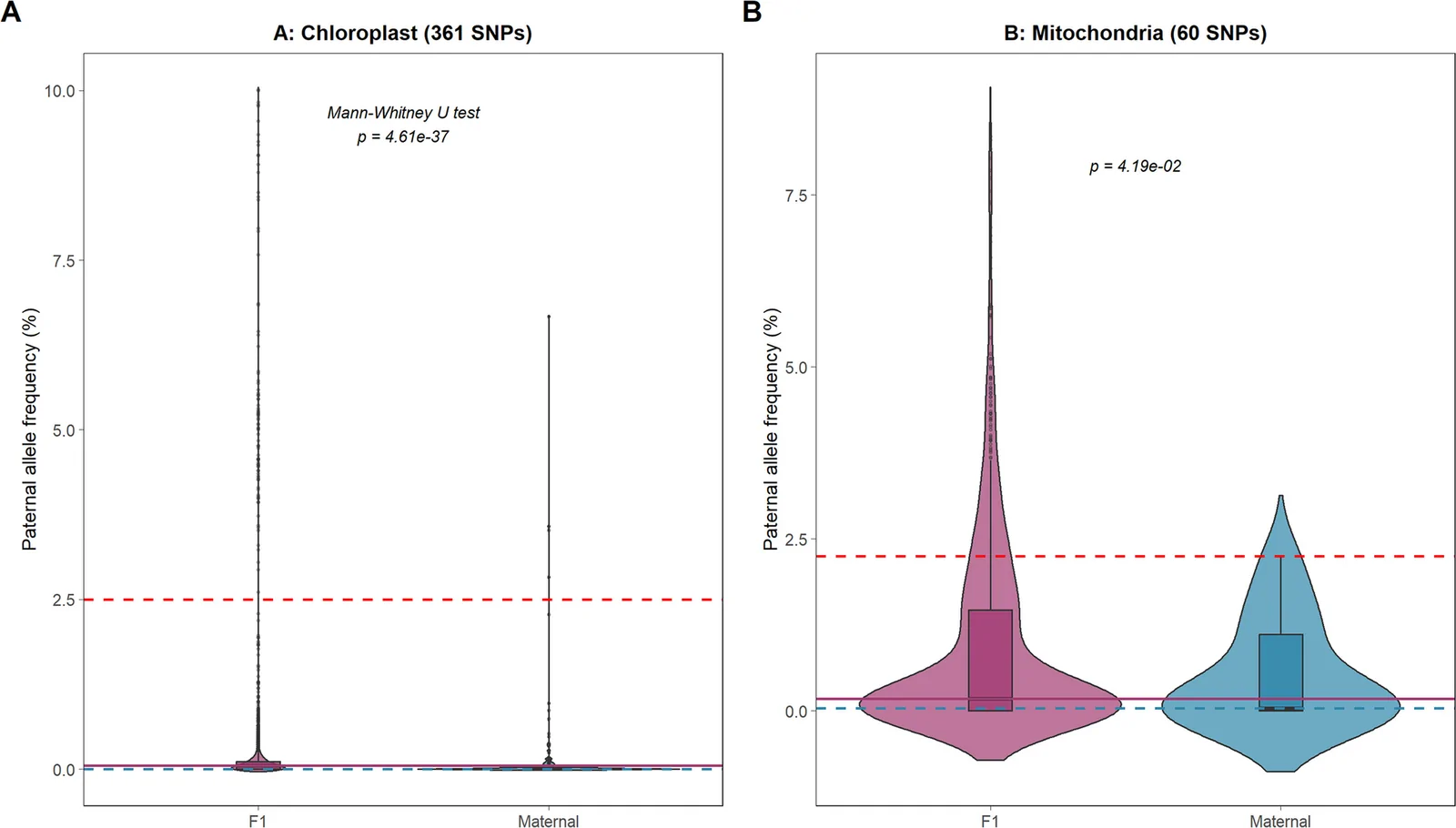
Quantitative evidence of strict maternal inheritance. **a** Chloroplast (361 diagnostic SNPs). Violin plots comparing paternal-type allele frequencies in the maternal background (blue) and F₁ progeny (purple). Median values indicated by dashed (maternal) and solid (F₁) horizontal lines. Dashed red line: 99th percentile threshold (2.50%). Mann–Whitney *p* = 4.61 × 10⁻^3^⁷. **b** Mitochondria (60 diagnostic SNPs). Median maternal 0.04%, median F₁ 0.18%; *p* = 0.042. All values remain below the 2.25% threshold

We examined all 361 chloroplast and 60 mitochondrial diagnostic SNPs across 21 F₁ individuals (8,841 independent tests). At chloroplast loci, the median paternal allele frequency in F₁ (0.05%) was significantly lower than the maternal background median (0.00%; Mann–Whitney U test, *p* = 4.61 × 10⁻3⁷). At mitochondrial loci, the median in F₁ (0.18%) was marginally higher than that in the maternal background (0.04%; *p* = 0.042) (Fig. 3a, b). Critically, all observed values remained below the empirical thresholds (2.50% and 2.25%, respectively).

### The elevated mitochondrial signal reflects maternal heteroplasmy, not paternal leakage

**Fig. 4:**
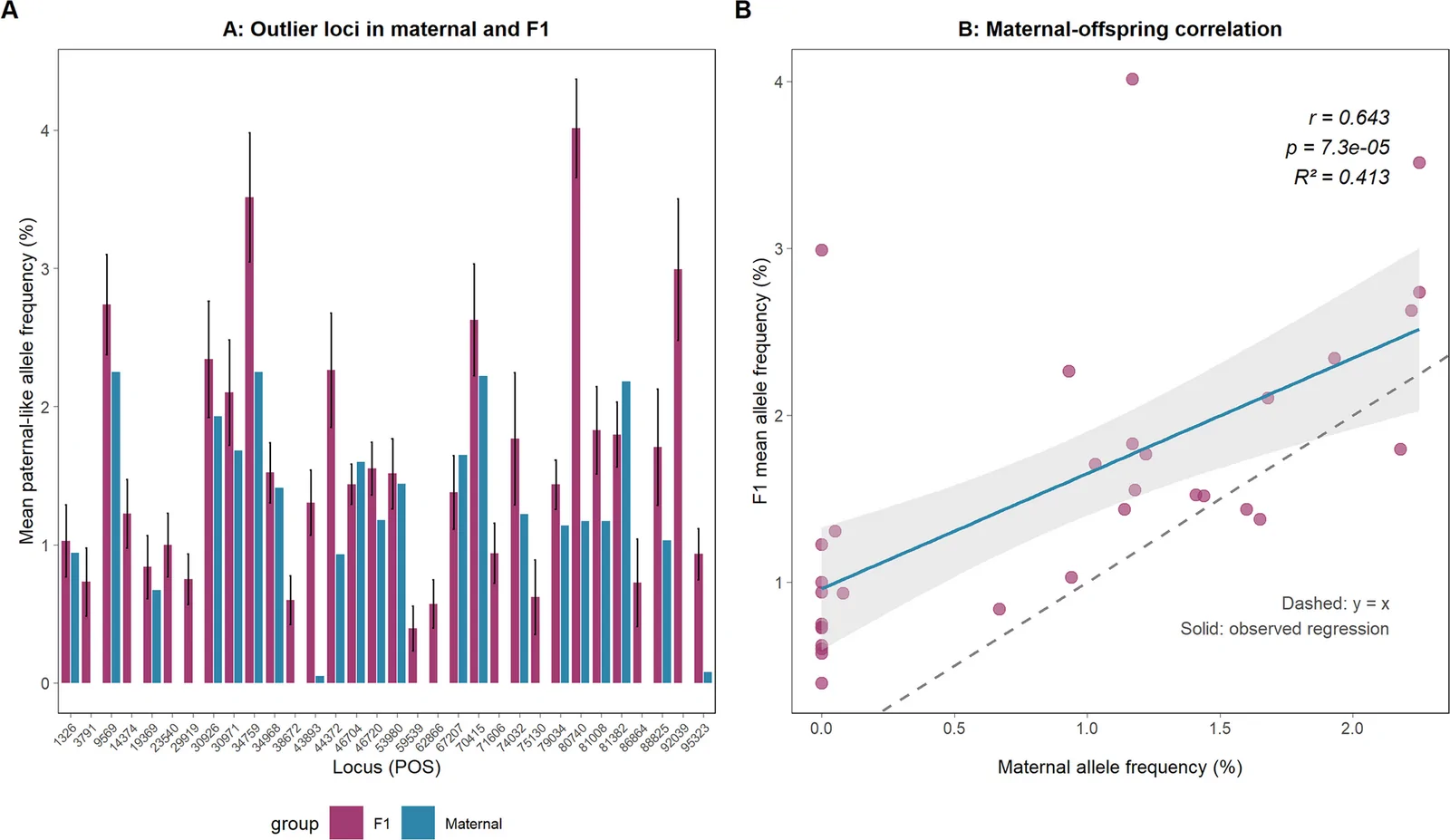
Stable maternal inheritance of mitochondrial heteroplasmy. **a** Mean paternal-type allele frequencies at 32 outlier loci where F₁ values exceed maternal background levels. Blue bars: maternal ma1; purple bars: mean of 21 F₁ individuals; error bars: SEM. **b** Scatter plot of maternal vs. mean F₁ allele frequency at the same 32 loci. Pearson's *r* = 0.643, *p* = 7.26 × 10⁻^5^; regression line (solid): y = 0.82x + 0.56, R^2^ = 0.413; dashed diagonal: y = x (perfect inheritance). The strong positive correlation demonstrates that F₁ signals originate from pre-existing maternal heteroplasmy, not paternal leakage

The slight mitochondrial elevation was attributable to 32 loci (out of 60) where F₁ allele frequencies exceeded the corresponding maternal background levels. These 32 sites showed a strong positive correlation between maternal and F₁ signals (Pearson's *r* = 0.643, *p* = 7.26 × 10⁻5), with maternal heteroplasmy explaining 41.3% of F₁ variation (R2 = 0.413) (Fig. 4a, b). These loci included lower-frequency variants that became discernible only through cross-generational comparison, beyond the 17 high-confidence heteroplasmic sites (AF > 5%) detected in the maternal parent alone. This finding has important methodological implications: without cross-generational correlation analysis, these 32 mitochondrial loci could have been misclassified as paternal leakage events. Instead, the elevated mitochondrial signal represents faithful inheritance of pre-existing maternal heteroplasmy.

### Conservative estimate of paternal leakage frequency

Across 8,841 independent tests, we observed zero paternal-specific alleles exceeding the noise thresholds. The upper 95% confidence bound for true paternal leakage was 0.034% (3/8,841). Applying a conservative approach that accommodates variable sequencing depth, uncorrected technical artifacts, and cross-study comparability [3, 15], we report the paternal leakage frequency as ≤ 0.1%. Both organelles exhibit consistently strict maternal inheritance with no detectable difference between chloroplasts and mitochondria.

## Discussion

The organelle genomes of *B. argenteum* align closely with the conserved architecture documented across mosses. Most moss mitochondrial genomes range from ~ 100 to 110 kb [44–47, 51], and a phylogenomic survey of 12 species revealed that mitogenome architecture has remained largely frozen for 350 million years, with only two independent gene losses and one gene relocation [47]. Similarly, moss plastomes cluster at 120–140 kb with the canonical quadripartite structure and 81–83 protein-coding genes [44, 48–50]. The *B. argenteum* chloroplast genome (122,995 bp, 123 genes) and mitochondrial genome (101,373 bp, 65 genes) conform fully to these patterns, reinforcing the picture of exceptional structural stasis in bryophyte organelle genomes.

Prior work established that bryophyte organelle inheritance is qualitatively maternal: *Pellia borealis* (liverwort) [21], *Rhizomnium pseudopunctatum* [22], *Ceratodon purpureus* [23], and *Sphagnum* [24] each demonstrated maternal transmission of chloroplasts and/or mitochondria. However, the absence of paternal leakage events in these small sample sizes cannot exclude low-frequency leakage—a distinction that has become increasingly important as quantitative estimates in angiosperms (0.18% in tobacco [4]) and animals (one event per 385 transmissions in chicken [8]) demonstrate that paternal leakage, though rare, is widespread.

Our study fills this quantitative gap. By leveraging 464 chloroplast and 96 mitochondrial diagnostic sites between intercontinental parental genotypes (including 361 cp and 60 mt SNPs suitable for quantitative allele-frequency analysis), and performing 8,841 independent allele frequency tests across 21 F₁ progeny, we established an upper bound of ≤ 0.1% for paternal leakage in *B. argenteum* (95% confidence limit: 0.034%). PCA of all 421 diagnostic SNPs confirmed that F₁ individuals are indistinguishable from the maternal parent (PC1 = 98.1%; Fig. 1c). To our knowledge, this is the first quantitative estimate of paternal leakage frequency in any bryophyte, and one of the most comprehensive in any non-seed plant.

The detection thresholds derived from the maternal background were notably consistent between organelle genomes (2.50% for chloroplasts, 2.25% for mitochondria), suggesting that at ~ 1000 × sequencing depth, a practical detection floor of ~ 2.5% for paternal-type allele frequencies is achievable. This provides a useful benchmark for designing future studies: achieving comparable sensitivity requires sequencing depth sufficient to distinguish the 99th percentile of maternal background noise from true paternal leakage signals.

A noteworthy finding is the systematic elevation of mitochondrial paternal-type allele frequencies in F₁ progeny (median 0.18% vs. 0.04% in the maternal background, *p* = 0.042), which at face value could suggest low-level paternal leakage. However, the strong maternal–F₁ correlation (*r* = 0.643, R2 = 0.413) at the 32 contributing loci demonstrates that these signals originate from pre-existing maternal heteroplasmy rather than paternal transmission. This observation echoes findings in human mtDNA studies, where apparent biparental inheritance signals were traced to nuclear-mitochondrial segments (NUMTs) or maternal heteroplasmy [14, 15, 17].

By contrast, chloroplast signals showed the opposite pattern: the median paternal-type allele frequency in F₁ (0.05%) was near zero and significantly lower than the already negligible maternal background (0.00%; *p* = 4.61 × 10⁻3⁷), reflecting the near-complete absence of both maternal heteroplasmy and paternal leakage at chloroplast loci. The cp F₁ median being slightly above zero (0.05%) while the maternal median is exactly zero likely reflects low-level technical noise rather than biological signal. This asymmetry between the two organelle genomes—detectable maternal heteroplasmy in mitochondria but not in chloroplasts—warrants further investigation and may reflect differences in organelle copy number, mutation rate, or the efficiency of heteroplasmy sorting during gametogenesis.

Our results highlight a general methodological principle for organelle inheritance studies: cross-generational correlation analysis is essential for distinguishing maternal heteroplasmy from authentic paternal leakage. Single-generation comparisons of allele frequencies between parents and progeny are insufficient. The three-stage framework employed here—(1) empirical noise characterization via self-mapping and cross-mapping, (2) percentile-based thresholding of the maternal background at diagnostic sites, and (3) maternal–F₁ correlation analysis—provides a template applicable to future studies in other non-model organisms.

Several caveats should be considered. First, our sample size—seven capsules and 21 F₁ gametophytes from a single intraspecific cross—is modest. While 8,841 independent tests provide substantial statistical power for detecting paternal leakage above ~ 0.03%, rarer events could escape detection. Second, we examined only one cross combination (Antarctic maternal × Beijing paternal); organelle transmission patterns could vary with different parental genotypes or environmental conditions. Third, despite > 1000 × sequencing depth, ultra-rare paternal leakage events at frequencies below 0.01% would require deeper sequencing or digital droplet PCR. Fourth, our analysis was restricted to diagnostic SNP sites; paternal leakage at conserved sites would not be detected. Fifth, the study examined a single species, and the generality of these findings across the diverse bryophyte lineage remains to be established. Future studies incorporating multiple cross combinations, larger progeny arrays, environmental perturbations, and complementary detection methods would further strengthen the quantitative framework established here.

## Conclusions

This study provides the first quantitative assessment of paternal leakage frequency in a bryophyte. Using comprehensive organelle genome assemblies, controlled intraspecific crosses, and deep sequencing of 21 F₁ progeny, we demonstrate strict maternal inheritance of chloroplasts and mitochondria in *B. argenteum* (paternal leakage ≤ 0.1%; 95% CI upper bound 0.034%). PCA of 421 diagnostic SNPs confirmed that all F₁ individuals cluster tightly with the maternal parent (PC1 = 98.1% of variance). The slight elevation of mitochondrial paternal-type signals in F₁ progeny was resolved as faithful transmission of maternal heteroplasmy rather than paternal leakage (Pearson's r = 0.643 between maternal and F₁ allele frequencies), underscoring the importance of cross-generational correlation analysis in organelle inheritance studies. The analytical framework presented here—empirical noise thresholding, maternal heteroplasmy correction, and binomial confidence bounding—is broadly applicable and offers a methodological template for future quantitative studies of organelle transmission in non-seed plants.

## Supplementary Information

Supplementary Material 1.

## Data Availability

The whole-genome sequencing data are openly available in Science Data Bank at https://doi.org/10.57760/sciencedb.23476, https://doi.org/10.57760/sciencedb.34716 and https://doi.org/10.57760/sciencedb.34648. The complete chloroplast and mitochondrial genome sequences generated in this study have been deposited in GenBank under accession numbers PZ276158, PZ276159, PZ276160, and PZ276161. The datasets generated during and/or analysed during the current study are available from the corresponding author on reasonable request.

## Abbreviations

SNP: Single nucleotide polymorphism
indel: Insertion/deletion
NUMT: Nuclear mitochondrial pseudogene
ddPCR: Digital droplet PCR
CTAB: Cetyltrimethylammonium bromide
MS: Murashige and Skoog
LSC: Large single copy
SSC: Small single copy
IR: Inverted repeat
AF: Allele frequency
PCA: Principal component analysis
