# Supplementary material for: Strict maternal inheritance of chloroplast and mitochondrial genomes in Bryum argenteum
Source: BMC Plant Biol. 2026 Jul 31;26:1837. doi: 10.1186/s12870-026-09627-y (PMC13711858; doi:10.1186/s12870-026-09627-y)
Supplement: Supplementary file 1 [file 12870_2026_9627_MOESM1_ESM.docx]

**Supplementary Table S1.** Summary of maternal organelle haplotype matching across 21 F₁ progeny. For each F₁ individual, the number and percentage of diagnostic chloroplast (out of 361) and mitochondrial (out of 60) SNP sites with paternal allele frequencies below the maternal 99th percentile threshold are listed.

| Supplementary Table S1 | | | | |
| --- | --- | --- | --- | --- |
| Sample | Pt_n_SNPs | Pt_match_maternal_pct | Mt_n_SNPs | Mt_match_maternal_pct |
| A21 | 357 | 98.88 | 54 | 88.89 |
| A41 | 357 | 98.88 | 59 | 84.75 |
| B22 | 361 | 98.61 | 58 | 79.31 |
| C12 | 361 | 98.89 | 58 | 84.48 |
| C13 | 361 | 98.89 | 58 | 94.83 |
| C43 | 361 | 98.89 | 58 | 86.21 |
| D11 | 361 | 98.89 | 58 | 86.21 |
| D21 | 361 | 98.89 | 58 | 93.1 |
| D22 | 361 | 98.89 | 58 | 93.1 |
| D33 | 361 | 98.89 | 58 | 94.83 |
| D41 | 361 | 98.89 | 58 | 82.76 |
| D43 | 361 | 98.89 | 58 | 91.38 |
| E14 | 361 | 98.61 | 58 | 74.14 |
| E23 | 361 | 98.89 | 58 | 87.93 |
| E34 | 361 | 98.61 | 58 | 72.41 |
| F14 | 361 | 98.89 | 58 | 79.31 |
| F41 | 361 | 98.89 | 58 | 91.38 |
| G11 | 361 | 98.89 | 58 | 87.93 |
| M21 | 361 | 98.89 | 58 | 82.76 |
| N11 | 361 | 98.61 | 58 | 74.14 |
| T12 | 361 | 98.89 | 58 | 72.41 |
| ma1 (maternal) | 361 | 100 | 58 | 100 |
